# Abdominal Leak Point Pressures During Cough and Valsalva: What Do the Different Maneuvers Hide?

**DOI:** 10.7759/cureus.104168

**Published:** 2026-02-24

**Authors:** Carolina Veiga e Moura, Ana Lopes, Bercina Candoso

**Affiliations:** 1 Obstetrics and Gynecology, Unidade Local de Saúde de Santo António, Porto, PRT

**Keywords:** abdominal leak point pressure, pelvic floor, sphincteric insufficiency, stress urinary incontinence, urethral continence, urodynamic study

## Abstract

This study analyzes abdominal leak point pressures (ALPP) during Valsalva and cough maneuvers in patients with stress urinary incontinence (SUI) (pure or mixed), with the aim of clarifying the different psychopathological mechanisms of urethral incontinence behind leakage with each.

This is a retrospective study of women with stress or mixed incontinence submitted to urodynamic testing with ALPP in 2024. Patients were allocated in mutually exclusive groups: group 1: Valsalva and cough leakage (n=57); group 2: cough leakage (n=20); and group 3: Valsalva leakage (n=10). A comparative analysis using IBM SPSS Statistics for Windows, Version 28.0 (IBM Corp., Armonk, New York, United States) was performed.

Average ALPP was higher in group 2 (113.8±44.3 cmH2O). ALPP was below 60 cmH2O in 37%, 10%, and 20% of the patients in groups 1, 2, and 3, respectively (p=0.06). Women in group 3 were significantly younger (p=0.001) and pre-menopausal (p=0.02).

In a subgroup analysis in group 1, only 31% of the patients had Valsalva leak point pressure (VLPP) superior to cough leak point pressure (CLPP). Body mass index (BMI) was significantly variable between subgroups, being higher in patients whose VLPP was superior to CLPP (p=0.01). Similarly, in a subgroup analysis in group 2, the pressure generated with Valsalva was superior to CLPP only in 25% of the cases. BMI was significantly higher in patients whose Valsalva-generated pressure was superior to CLPP (p=0.004).

Cough and Valsalva maneuver are both equally and individually relevant in the setting of ALPP, but not equivalent. Our findings support that each maneuver might be associated with different underlying mechanisms for SUI and both should be performed during ALPP.

## Introduction

Increased abdominal pressure does not cause leakage in a functional and anatomically normal urethra [1]. Urethral continence mechanisms are multiple, and failure in one of them can lead to leakage and, therefore, incontinence. Even though several tests of urethral function have been proposed, abdominal leak point pressure (ALPP), the lowest recorded pressure that results in urinary leakage, has been considered the best measure of urethral sphincter strength [2]. However, the International Continence Society (ICS) Standardization of Terminology did not include leak point pressures. The absence of standardized performance methodology poses challenges to both the reproducibility of this test and the interpretation of its results.

ALPP is usually performed through maneuvers that increase intra-abdominal pressure, such as coughing and straining (Valsalva). Despite some inter-patient variability in the performance of both tests (inability to generate adequate pressures due to anxiety, misunderstanding of instructions), not rarely different outcomes are obtained with different maneuvers. Other than studies focusing on the accuracy of each test, only a few authors have focused on the true significance of obtaining different leakage patterns with different maneuvers, both destined to increase intra-abdominal pressure. Suggestions have been made that the continence control system reacts differently to cough and Valsalva. Indeed, while straining with Valsalva requires pelvic floor relaxation, coughing leads to a rise in the stiffness of the suburethral tissue, attributed to a reflex contraction of the striated levator ani muscles [3-5]. Clarification of the cough and straining difference points might allow us to extrapolate information regarding the dynamic operation of the urethral continence mechanisms.

This study focuses on the information retrieved by Valsalva and cough maneuvers as well as on a careful interpretation of different patterns of response in patients with a history of stress urinary incontinence (SUI). Our primary aim was to ascertain phenotypic differences between patients with distinctive responses to intra-abdominal raising maneuvers that could endorse our primary mechanistic hypothesis: that leakage with Valsalva or cough is associated with different psychopathological mechanisms of urethral incontinence. 

## Materials and methods

Subjects and study design

**Figure 1:**
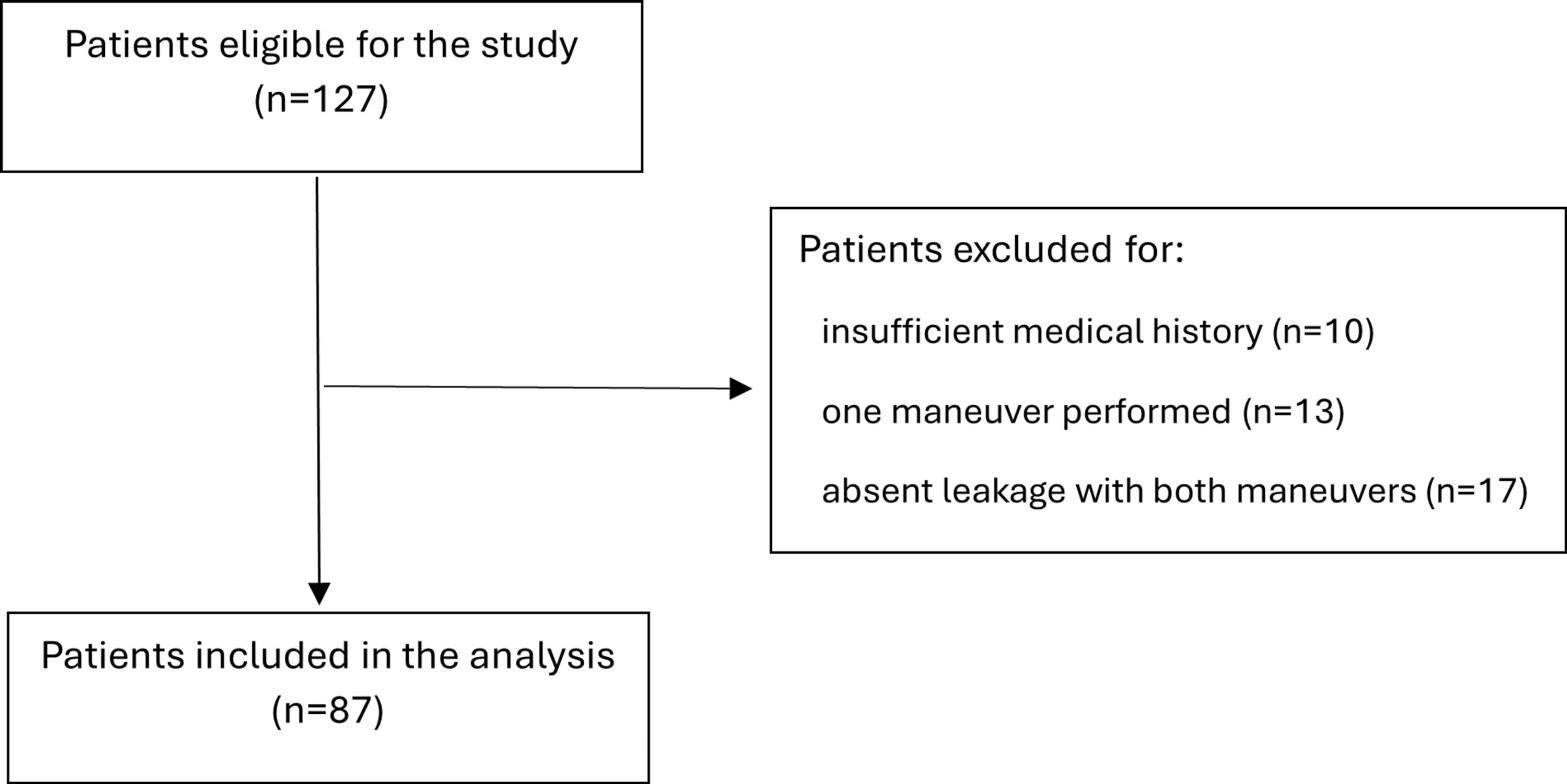
Flowchart of patient selection

Our target population consisted of women with SUI (pure or mixed) who had been submitted to urodynamics with leak point pressure studies. Our sample frame ended up consisting of women with a history of stress or mixed incontinence, submitted to urodynamic testing with leak point pressure during the year 2024 (n=127). Patients were retrospectively analyzed, and clinical data were retrieved from digital platforms, with focus on the following variables of interest: type of incontinence, age, body mass index (BMI), parity, menopause, medical history (diabetes mellitus, neuropathy, asthma), and presence of anterior organ prolapse on physical examination. SUI was defined as any persistent involuntary loss of urine on effort or physical exertion; mixed urinary incontinence was defined as the persistent complaint of involuntary leakage associated with both urgency and exertion. BMI was calculated from a metric formula according to the patient's weight and height. Menopause was assumed based on the absence of menstrual period for 12 months in women without contraception. Anterior organ prolapse was defined on physical examination according to the Pelvic Organ Prolapse Quantification (POP-Q) staging. Women were excluded if they complied with one or more of the following criteria: only one maneuver (Valsalva leak point pressure (VLPP) or cough leak point pressure (CLPP)) performed or absence of leak with both maneuvers and insufficient medical history. A total of 40 women were excluded after the application of the exclusion criteria (see Figure 1).

Urodynamic study

No preparation was required for our patients. After uroflowmetry without residue confirmation, patients were placed in the upright sitting position. A 7fr 2/3 way bladder catheter and a 6fr open balloon rectal catheter were purged, connected to the transducer, and posteriorly placed on the patient. Zeroing to atmosphere was performed, and catheter lines were filled; troubleshooting was performed if needed. Vesical and abdominal pressures were verified, and the correct placement of catheters was assured by asking the patients to cough and assessing transmissibility. After that, retrograde cystometry with double-distilled water was performed at a 50 mL/min rate up to volumes between 150 and 200 cc; after volume achievement, the vesical catheter was removed so as to exclude false elevations in ALPP due to intraluminal catheterization. A fluid-filled system was used for pressure measurement. The patients were asked to cough and strain two times in a row with the following verbal commands: "Please cough twice" (if insufficient, "Please cough harder") and "Please take a deep breath, hold it in, and push as if you are delivering a baby or passing hard stools". Patients with anterior compartment pelvic organ prolapse underwent the test with manual prolapse reduction. CLPP and VLPP were considered as the lowest abdominal pressures, measured through the in-place rectal catheter, which caused directly visible leakage through the external meatus in the absence of a detrusor contraction during the respective maneuvers. In case of multiple leaks, we selected the lowest value, so as to better reflect urethral resistance, instead of using the average. All the tests were performed in the presence of two clinicians, with an interobserver agreement on leakage of 93.1%. At the end, patients were asked to empty their bladder, after which the rectal catheter was removed. No post-study procedures were undertaken. All patients had a follow-up urogynecology appointment booked in less than two months of distance.

Subgroup and statistical analysis

A total of 87 patients were obtained. Patients were divided according to the leakage pattern: group 1 consisted of patients with leakage with both Valsalva and cough (n=57), group 2 contained patients with leakage only with cough (n=20), and group 3 consisted of patients with leakage only with Valsalva (n=10). A comparative analysis using IBM SPSS Statistics for Windows, Version 28.0 (IBM Corp., Armonk, New York, United States) was performed considering the following variables: age, BMI, parity, history of diabetes mellitus or neuropathy, and presence of anterior organ prolapse. The chi-squared test or Fisher's exact test was used as appropriate to compare the categorical variables. After assessing for normality, quantitative data were compared using independent samples t-tests/ANOVA tests for parametric variables or Mann-Whitney/Kruskal-Wallis tests for non-parametric ones. The level of statistical significance was set with a p-value of ≤0.05.

## Results

Demographic characteristics

Our patients' average age at examination was 56.2±12.9 years, with 69% of our sample being post-menopausal women. Average BMI was 29.4±5.5 kg/m^2^. Twenty percent of the patients had diabetes mellitus, 7% asthma, and almost 5% either central or peripheral neuropathy. Almost 42% of the patients complained of SUI, and the remaining had mixed incontinence. From the latter, 25% were under overactive bladder pharmacological treatment prior to the urodynamic study.

Patients who leaked under Valsalva (groups 1 and 3) had mean Valsalva leak pressures of 79±26 cmH2O. From the ones who leaked with cough (groups 1 and 2), the mean cough pressure at leakage was 99.4±37.8 cmH2O.

The average ALPP was 71.6±23.2 cmH2O, 113.8±44.3 cmH2O, and 85.9±39.1 cmH2O in groups 1, 2, and 3, respectively. According to the lowest pressures at leakage, as suggested by McGuire et al. [6], women from distinct groups were allocated to either one of the two groups based on the aetiology of SUI: urethral support failure and equivocal or urethral sphincteric failure (Table 1). Indeed, urethral sphincteric failure was assumed in 37%, 10%, and 20% of the patients in groups 1, 2, and 3, respectively (p=0.06). When excluding equivocal results, sphincteric failure was assumed in 56%, 14%, and 40% of the patients in groups 1, 2, and 3, respectively (p=0.02).

**Table 1:** Comparative analysis of ALPP between groups ALPP: abdominal leak point pressures

ALPP (cmH2O)	Group 1 (n, %)	Group 2 (n, %)	Group 3 (n, %)
<60	21 (37)	2 (10)	2 (20)
60-90	20 (35)	6 (30)	3 (30)
>90	16 (28)	12 (60)	5 (50)

Subgroup analysis: groups 1, 2, and 3

A comparative analysis between groups was conducted regarding the abovementioned variables, as shown in Table 2. The average age was significantly different between groups, with women in group 3 being significantly younger (p=0.001) and therefore non-menopausal (p=0.015). Identification of detrusor hyperactivity was also significantly different between groups (p=0.05), with it being more prevalent in group 2. None of the other variables showed statistical significance.

**Table 2:** Comparative analysis between the three groups BMI: body mass index

	Group 1	Group 2	Group 3	P-value
Age (y)	59.6±12.3	59.6±11.5	46.0±12.2	0.001
BMI	29.1±6.1	31.5±5.1	28.4±2.2	0.48
Parity (n)	1.8±1.3	1.7±0.9	1.63±0.7	0.81
Vaginal deliveries (n)	1.6±1.2	1.2±1.0	0.88±0.6	0.60
Menopausal (%)	79	55	40	0.02
Diabetes mellitus (%)	23	10	20	0.49
Neuropathy (%)	5	0	10	0.45
Asthma (%)	5	5	20	0.24
Anterior compartment pelvic organ prolapse (%)	19	20	22	0.98
Detrusor hyperactivity (%)	5	10	3	0.05

In a multiple linear regression analysis, age, BMI, and detrusor hyperactivity were predictive of leakage with either one or both maneuvers (F(3,43)=3.997; p=0.01; R2=0.218). Nevertheless, when looking at individual variables, only age was statistically significant (p=0.005).

The median volume instillation was 200 mL in all groups, with no significant differences registered between them regarding volume distribution (p=0.33). No correlation was found between instilled volume and CLPP (r=0.181; n=76; p=0.12), VLPP (r=0.055; n=66; p=0.66), or inferred aetiology of SUI as defined by McGuire et al. [6] (r=0.131; n=87; p=0.23).

We opted to look further into each group.

Subgroup Analysis: Group 1

In group 1, the mean VLPP and CLPP were 77.3±23.8 cmH2O and 94.6±34.5 cmH2O, respectively. Thirty-eight patients had CLPP superior to VLPP, with the remaining 18 showing the opposite. A subgroup analysis was undertaken in group 1, as seen in Table 3. BMI was the only statistically significant variable between groups, being higher in patients whose VLPP was superior to CLPP.

**Table 3:** Comparative analysis between subgroups inserted in group 1 VLPP: Valsalva leak point pressure; CLPP: cough leak point pressure; BMI: body mass index

	VLPP > CLPP	CLPP > VLPP	P-value
Age (y)	61.5±12.7	58.8±12.2	0.46
BMI	32.9±7.6	27±4.1	0.01
Parity (n)	2.2±1.4	1.7±1.1	0.20
Vaginal deliveries (n)	1.8±1.3	1.5±1.2	0.30
Menopausal (n)	30	14	0.92
Diabetes mellitus (n)	6	5	0.40
Neuropathy (n)	4	1	0.20
Asthma (n)	2	1	0.98

Subgroup Analysis: Group 2

In group 2, despite the absence of leakage, the Valsalva maneuver generated intra-abdominal average pressures of 95.0±27.7 cmH2O. In five patients, CLPP was inferior to the pressure generated with Valsalva. A subgroup analysis was also led in group 2, comparing patients whose Valsalva pressure in the absence of leakage was superior to that generated by CLPP versus the opposite, as seen in Table 4.

**Table 4:** Comparative analysis between subgroups inserted in group 2 VLPP: Valsalva leak point pressure; CLPP: cough leak point pressure; BMI: body mass index

	VLPP > CLPP	VLPP < CLPP	P-value
Age (y)	49.3±7.0	52.9±13.1	0.54
BMI	37.5±0.71	29.5±4.1	0.004
Parity (n)	2.2±0.84	1.54±0.88	0.17
Vaginal deliveries (n)	1.2±1.1	1.1±1.1	0.83
Menopausal (n)	3	8	0.60
Diabetes mellitus (n)	0	2	0.69
Asthma (n)	0	1	0.82
Anterior compartment pelvic organ prolapse (n)	1	3	0.81

BMI was the only statistically significant variable between groups, higher in patients whose Valsalva-generated pressure was superior to CLPP.

Subgroup Analysis: Group 3

In group 3, despite the absence of leakage, cough generated intra-abdominal average pressures of 121.8±34.7 cmH2O. The Valsalva-generated intra-abdominal pressures were inferior to CLPP in all patients; therefore, no subgroup analysis was performed.

## Discussion

The goal of our study was to assess the intricacies of each maneuver during the ALPP study. Coughing is a widely known mechanism for leakage in everyday life; however, due to the acute rise in pressure, the exact value at which it occurs can result from the assumption of leakage at the peak of the cough spike. Indeed, whereas some authors have recommended that Valsalva should be performed prior to cough, given its higher accuracy [7], others have suggested them as being equivalent [8,9]. Nonetheless, we put our focus on the significance of each maneuver instead of on its accuracy as a diagnostic marker of SUI.

When comparing the three groups of patients, age, menopause, and concomitant detrusor hyperactivity were the only significant variables between groups. When strictly opposing groups 2 and 3, women with cough leakage are tendentially but not significantly more obese and more parous and have more previous vaginal deliveries. The association of cough leakage with known risk factors to pelvic floor weakness might be explained by the hypothesis that leakage to cough is, indeed, a marker of pelvic floor weakness. On the contrary, women in group 3 had a higher, although not significant, prevalence of neuropathy and diabetes mellitus, supporting the hypothesis that Valsalva leakage might be associated with a dysfunctional pelvic floor neuromuscular component. Indeed, this could also explain a significantly higher incidence of concomitant detrusor hyperactivity in the spectrum of overactive bladder disorder.

In a similar fashion to reports in literature, the vast majority of our patients, regardless of the group, had lower leak pressures with Valsalva in comparison to those generated with cough. This can be explained considering the physiology of the mechanisms of continence: whereas VLPP represents the intra-abdominal pressure that exceeds the intrinsic urethral resistance, CLPP adds to that value of pressure the one generated by a counteracting suburethral tissue force, mediated by the pelvic floor [3-5]. In group 1, focusing on the minority of patients whose VLPP was superior to CLPP, the only statistically significant variable that distinguished this subgroup from the majority was a higher average BMI. Nonetheless, women with higher VLPP were tendentially older, with more previous pregnancies and/or vaginal deliveries and increased prevalence of neuropathy. Despite our small sample size, this finding might suggest that, in this minority of patients, cough is not able to generate an effective activation of the levator ani muscles, which can be attributed to either an impaired muscular function (to which obesity and parity are known risk factors) or a blunt nerve supply. To further enhance this hypothesis, in 25% of the patients from group 2, pressures generated with the Valsalva maneuver without leakage were higher than those referring to the CLPP. Indeed, in these patients, in comparison to the remaining 75%, obesity was also significantly more prevalent. Despite non-significantly, women in that group were also older and tendentially multiparous.

Several classifications for SUI have been suggested. We focused on the one proposed by McGuire et al. [6] and hypothesized that Valsalva and cough maneuvers could be individually associated with different dichotomic mechanisms of SUI: urethral support failure and urethral sphincteric failure. Indeed, in our sample, urethral sphincteric failure was assumed in 37%, 10%, and 20% of the patients in groups 1, 2, and 3, respectively (p=0.06). When excluding equivocal values of ALPP (60-90 mmHg), groups 1, 2, and 3 were allocated to sphincteric failure in 56%, 14%, and 40% of the cases, respectively, with significant differences between groups (p=0.02). Considering our results, sphincter failure was least associated with leakage with cough; and, as seen previously, at the same time, cough leakage was most incident in patients with risk factors associated with insufficient pelvic floor. Our primary hypothesis was that leakage with Valsalva or cough might be associated with different psychopathological mechanisms of urethral incontinence. Indeed, similar to previously proposed interpretations of the latter, our findings could endorse the idea that leakage with the cough maneuver is most accurate and, consequently, possibly representative of insufficient pelvic floor. Whereas our findings corroborate this hypothesis, the absence of concomitant complementary urodynamic parameters, such as videourodynamics, electromyography of the pelvic floor muscles, and/or urethral profilometry, does not allow us to draw definite conclusions. Nevertheless, a quite considerable proportion of patients in group 3 had leak point pressure compatible with urethral support failure, which precludes us from drawing conclusions as to the association of the Valsalva maneuver with sphincteric failure. Although almost half of the patients in group 3 had ALPP inferior to 90 cmH2O, conclusions cannot be drawn as to its sensitivity in assessing sphincteric insufficiency. Indeed, a relevant proportion of patients showed equivocal results, which makes it entirely difficult to reach conclusions.

Strengths and limitations

ALPP's performance methodology hasn't been standardized, and its value in assessing urethral continence isn't consensual throughout the literature. Nevertheless, we believe it to be a harmless test, which can provide additional information in women undergoing filling cystometry and pressure-flow studies. In our sample, the performance of only one intra-abdominal raising pressure maneuver led to the exclusion of 13 patients from our sample. The absence of accompanying video in the urodynamic study and/or additional urethral profilometry tests is an undeniable limitation in our setting, as it could have further elucidated to us the impairment of mechanisms of continence during each maneuver.

Some reports claim volumes between 250 mL and 300 mL as the most accurate in distinguishing sphincter failure from pelvic floor insufficiency. In our sample, infused volumes were individualized according to maximum cystometric and functional capacity, which led to volumes between 150 mL and 200 mL. While these volumes might increase our false-negative results, our general practice has led to the belief that these are often enough to obtain a valid study, at the same time avoiding detrusor hyperactivity that might compromise the results.

Perhaps the most striking limitation of our study is our short sample of patients and the absence of power analysis, which limits our hypothesis to exploratory rather than conclusive. Moreover, in our subgroup analysis, adjustment for multiple comparisons wasn't performed, which should be taken into account when looking at results, given the risk of false positivity. Additionally, the occasional absence of reproducibility of the urodynamic study might not allow the extrapolation of results to larger groups.

Previous reports in the literature exclude certain comorbidities in the evaluation of ALPP. We opted to include them (diabetes mellitus, neuropathy, asthma), as we believe it not only provided answers to our hypotheses but also strengthened them; we focused solely on the stress incontinence component. Furthermore, we opted not to exclude patients with mixed incontinence symptoms. Indeed, in literature, the study population usually consists of patients with pure stress incontinence, excluding patients with concomitant detrusor overactivity or urgency symptoms. We believe this occurs due to the fear of generating an abnormal detrusor contraction with the Valsalva technique. Nevertheless, the definition of leak point pressure precludes leakage in the presence of a detrusor contraction during this maneuver. Moreover, both cough and Valsalva can lead to inhibited detrusor contractions. Whereas that might constitute a difficulty in the continuity of the urodynamic study, we believe it is the reporting clinician's responsibility to duly interpret it.

Our ALPP was performed without a vesical catheter, which might have strengthened the accuracy of our results. Indeed, its presence might have led to a falsely elevated leak point pressure in some subset of patients, given the partial obstruction of the urethral lumen.

## Conclusions

This study supports the idea that cough and Valsalva maneuver are both equally and individually relevant in the setting of ALPP, despite not being equivalent. Indeed, our findings point towards the idea that each maneuver might be associated with different mechanisms for SUI and both should be performed in the assessment of women with SUI.

Increased knowledge on the individual application of each intra-abdominal raising maneuver could, in the future, lead to more accurate diagnosis of the underlying aetiology for SUI and, consequently, to better targeting of therapeutic options.
